# IGF1R is a mediator of sex-specific metabolism in mice: Effects of age and high-fat diet

**DOI:** 10.3389/fendo.2022.1033208

**Published:** 2022-10-20

**Authors:** Patricia Pérez-Matute, Icíar P. López, María Íñiguez, Emma Recio-Fernández, Raquel Torrens, Sergio Piñeiro-Hermida, Elvira Alfaro-Arnedo, Luong Chau, Christina Walz, Andreas Hoeflich, José A. Oteo, José G. Pichel

**Affiliations:** ^1^ Infectious Diseases, Microbiota and Metabolism Unit, Infectious Diseases Department, Center for Biomedical Research of La Rioja (CIBIR) -Hospital Universitario San Pedro, Logroño, Spain; ^2^ Lung Cancer and Respiratory Diseases Unit. Fundación Rioja Salud, Center for Biomedical Research of La Rioja (CIBIR), Logroño, Spain; ^3^ Miguel Servet Foundation-Navarra's Health Research Institute (IDISNA), Navarrabiomed Biomedical Research Center, Oncoimmunology Group, Pamplona, Spain; ^4^ Institute for Genome Biology, Research Institute for Farm Animal Biology (FBN), Dummerstorf, Germany; ^5^ Biomedical Research Networking Center on Respiratory Diseases (CIBERES), Instituto de Salud Carlos III (ISCIII), Madrid, Spain

**Keywords:** IGF1R, aging, insulin resistance, diet-induced obesity, fatty liver, inflammation, Insulin-like growth factors, sexual dimorphism

## Abstract

**Objective:**

We aimed to investigate the short and long-term metabolic consequences of IGF1R systemic gene deficiency in mice.

**Methods:**

*UBC-CreERT2, Igf1r^fl/fl^* mutant mice were used to suppress IGF1R signaling in adult tissues by inducing postnatal generalized *Igf1r* deletion with tamoxifen. Animals were analyzed at two different ages: *i*) 13-weeks old young mice, and *ii*) 12-months old middle-aged mice. In addition, the effects of 10 weeks-long high-fat diet (HFD) were investigated in middle-aged mice.

**Results:**

Young IGF1R-deficient mice were insulin-resistant, with high IGF1, growth hormone (GH) and IGFBP3, as well as low IGFBP2 circulating levels. Males also presented increased triglycerides in liver. In contrast, middle-aged mice did not clearly show all of these alterations, suggesting possible compensatory effects. Middle-aged IGF1R-deficient male mice were able to counteract the negative effects induced by aging and HFD in adiposity, inflammation and glucose metabolism. A metabolic sexual dimorphism dependent on IGF1R was observed, especially in middle-aged mice.

**Conclusions:**

These results demonstrate that IGF1R is involved in metabolic homeostasis, with effects modulated by diet-induced obesity and aging in a sex dependent manner. Thus, IGF1R deficiency in mice is proposed as a useful tool to understand metabolic alterations observed in patients with IGF1R gene deletions.

## Introduction

The insulin-like growth factor (IGF) system comprises two major ligands (IGF1 and IGF2), different receptors and IGF-binding proteins (IGFBPs), acting together to control multiple cellular functions such as growth, proliferation, differentiation, survival, adhesion, senescence, autophagy, epigenetics and migration in a variety of tissues and cells (1). A central member of this IGF system is the insulin-like growth factor 1 receptor (IGF1R), an ubiquitously expressed membrane-bound tyrosine kinase receptor with a high level of homology with the insulin receptor. IGF1R signaling results in the activation of different pathways mainly mitogen-activated protein kinases (MAPK) and phosphatidylinositol 3 kinase/Akt/mammalian target of rapamycin (mTOR). In addition, IGF1R signaling pathways, more complex than first thought, regulate essential biological/physiological functions including those related with metabolism, inflammation and aging (2–6).

Heterozygous mutations in the IGF1R gene, causing partial resistance to IGF1, are associated with several metabolic and endocrine alterations such as hypo/hyperlipidemia, hypothyroidism, diabetes/insulin resistance or even hypoglycaemia (7–9). These metabolic disorders associated with deletion of the IGF1R pathway revealed that IGF1/IGF1R signaling plays an important role in energy metabolism, muscle glucose uptake and also coordinates the responses to nutrient intake and the appropriate metabolic changes that enable cells to tolerate a variety of stressful stimuli (10–14). Insulin/IGF1 signaling also plays a crucial role in the control of brown and white fat development/functionality, thermogenesis and cold acclimation (11, 15–18).

Although disruption of IGF1R function is associated with several metabolic alterations, it also positively impacts life span by halting a variety of aging processes and aging-relevant metabolic features, delaying the progression of age-related diseases (19–23). Thus, long-term IGF1R blockade in adult mice could generate beneficial effects on aging-relevant metabolic features (22). However, there are some controversial results since *Igf1r* ^+/-^ mice do not appear to be an appropriate model of increased longevity and delayed aging (24). In addition, there are also controversies concerning how metabolic conditions in IGF1R compromised mice are dependent on sex and aging. Thus, whereas some authors do not find sex differences (22), others report that compromised IGF1R signaling has effects on aging in a sex dependent manner (20).

Here, we aimed to study how IGF1R generalized deficiency mediates metabolic alterations in male and female mice. The effects of age and a high-fat diet (HFD) were also addressed in our model.

## Materials and methods

### Generation of *Igf1r*-deficient mice

All animal procedures were carried out in accordance with the European Communities Council Directive on animal experiments (EU Directive 2010/63/EU) and were approved by the Institutional Animal Care & Use Committee (IACUC) from the Center for Biomedical Research of La Rioja (CIBIR), Spain (ref 03/12). All animals were bred and maintained under specific pathogen-free (SPF) conditions in laminar flow caging at the CIBIR animal facility.

*UBC-CreERT2; Igf1^rfl/fl^* mice were crossed with *Igf1r^fl/fl^* mice to directly generate descendants in equal proportions in the same litter. Tamoxifen (TMX) was daily administered for five consecutive days to four-week-old mice of both genotypes to induce a postnatal *Igf1r* gene conditional deletion (25). After TMX treatment, *UBC-CreERT2; Igf1r^Δ/Δ^ (CreERT2*;IGF1R-deficient) and *Igf1r^fl/fl^* (control) mice of both sexes were used for different experimental setups. All animals were fed with normal chow diet (Standard diet (STD), 801010 RM1A (P), UK). Young mice were sacrificed at the age of 13 weeks. Middle-aged mice were sacrificed at the age of twelve months. These animals received a second treatment of TMX when they were 8 months old to ensure complete deletion of *Igf1r* gene. Half of middle-aged mice were kept on standard diet (STD, RM1A (P); SDS, Essex, UK) (352 kcal/100g; 7.42% of kcal from fat) and the other half were changed to a HFD (60% of kcal from fat: D12492; Research Diets Inc., New Brunswick, NJ, USA) (524 kcal/100g) for the last 10 weeks before sacrifice. All animals were sacrificed after a 5-h fasting period. Blood and different organs (liver and perigonadal fat) were collected, weighted and immediately frozen in liquid nitrogen or fixed in 4% formaldehyde and paraffin embedded for subsequent histological studies.

### Biochemical parameters

Serum was collected from cardiac puncture after 5 hours of fasting. Serum levels of glucose were measured using an automatic biochemical analyzer (Cobas C711, Madrid, Spain). Insulin, gamma-glutamyltransferase (ƴGT), IGF1 and growth hormone (GH) were quantified by commercial ELISA Kits following manufacturer’s instructions (Merck-Millipore Corp., St. Charles, MA, USA; R&D Systems, Minneapolis, MN, USA and Cloud-Clone Corp, Katy, TX, USA). The intra- and inter-assay variation coefficients (CV) expressed as percentage are the following: for insulin: 3.7% and 10.5% respectively; ƴGT: <10% and <12%; GH: 2.6% and 4.2% and IGF1: 4.3% and 6% respectively. Insulin resistance was calculated using the homeostasis model assessment of insulin resistance (HOMA-IR) as previously described (26).

IGF-binding protein 2 (IGFBP2) was assessed in serum from mice at an age of 3 months by Western immuno-blotting by specific antiserum and recombinant human IGFBP2 as previously described (27). At the same age, IGF-binding protein 3 (IGFBP3) was quantified in serum by ELISA (Mouse/Rat IGFBP3 ELISA - E031, Mediagnost, Reutlingen, Germany) according to the instructions of the manufacturer. An intra-assay variance of 4.6% and an inter-assay variance of 9.06% was obtained in the IGFBP3 ELISA. At an age of one year, IGFBP2 and IGFBP3 were assessed by Western ligand blotting as described before (28).

### Histological immunostaining analyses

Following formalin fixation, perigonadal adipose tissue and liver were dehydrated and paraffin embedded. Tissue sections (3 μm-thick) were dewaxed and rehydrated by standard methods. For antigen retrieval, sections were immersed in a boiling solution of 1 mM EDTA (pH 9.0) for 15 min. Endogenous peroxide enzyme activity was blocked by 0.2% H_2_O_2_ for 15 min. After blocking with 4% goat serum in PBS-Triton (0.1%) and BSA (2%) for 1 h at room temperature slides were incubated at 4°C overnight with a primary rabbit polyclonal antibody anti Iba1 (ref. 019-19741, FUJIFILM Wako Pure Chemical; Osaka, Japan) at a 1:1000 dilution. Sections were treated with biotinylated anti-rabbit antibody (ref. BA-1000, Vector Laboratories; Burlingame, CA) at a 1:350 dilution, and visualized with avidin-biotin-peroxidase complex (Vector Elite ABC kit; Vector Laboratories) and diaminobenzidine substrate (K3468, FUJIFILM Wako Pure Chemical). Harris hematoxylin (Panreac; Barcelona, Spain) was used to counterstain the slides. Slides were observed under a light microscope (Nikon Instruments Inc.; Tokyo, Japan) (29, 30). Quantification of Iba1 positive cells and Iba1 crown-like structures were analyzed using a double-blinded protocol in male (*Igf1r^fl/fl^*, n = 4 and *CreERT2*, n = 4) and female (*Igf1r^fl/fl^*, n = 5 and *CreERT2*, n = 3) mice. Measurements were performed in 8-10 images selected in a random way (x200 original magnification, corresponding to approximately 3x10^5^ μm^2^) per section taken from two sections of each sample. Fiji opensource image processing software package v1.48r (http://fiji.sc) was used to quantify Iba1^+^ (percentage of DAB) areas (%) and total crown-like structures were counted on each image.

### Hepatic triglyceride content

To quantify liver triglyceride (TG) content, 150 mg of liver tissue were homogenized using Ultraturrax (IKA-Weke, Staufen, Germany) in 1.5 mL of buffer (150 mM NaCl, 0.1% Triton X-100 and 10 mM Tris pH 8) at 50°C. After centrifugation at 12000 g for 10 min, the supernatant was used for the measurement of TG levels, using an AutoAnalyzer as described elsewhere (31–33).

### RNA isolation, reverse transcription and qPCR

Total RNA was extracted from perigonadal fat pads and liver using TRIzol^®^ reagent (Invitrogen, Carlsbad, CA, USA) according to the manufacturer’s instructions. RNA concentration and purity was evaluated using a Nanodrop Spectrophotometer (ND-1000, Thermo Fisher Scientific, MA, USA). A 2 μg sample of total RNA was incubated with DNase (DNAse I Amplification Grade, Invitrogen, MA, USA) for 30 min at 37°C. RNA was then reverse-transcribed to cDNA using M-MLV reverse transcriptase (Invitrogen, Carlsbad, CA, USA). Sybr Premix Ex Taq (Takara Bio Inc., Shiga, Japan) and specific primers for insulin growth-factor (*Igf1*), IGF-binding protein 2 (*Igfbp2*), IGF-binding protein 3 (*Igfbp3*), Uncoupling protein 2 (*Ucp2*), Carnitine palmitoyltransferase 1A (*Cpt1a*), Fatty acid synthase (*Fas*), the macrophage receptor *F4/80*, the macrophage antigen *Cd68*, Tumor necrosis factor-alpha (*Tnfα*) and *β-actin* were used for quantitative PCR (q-PCR) (all from Sigma, St. Louis, MO, USA) (**Supplementary Table 1**). All procedures were performed according to the manufacturer’s instructions using the ABI PRISM 7300 (Applied Biosystems, Foster City, CA, USA). All PCR reactions were performed in triplicate, and *β-*actin was used to normalize gene expression. Ct values were generated by the ABI software. The relative expression level of each gene was calculated as 2^−ΔΔCt^ (34).

### Western blot analysis

Western blot analyses were performed in perigonadal fat depots and liver (young and middle-aged animals). Lysates were obtained by the addition of a RIPA buffer containing protease and phosphatase inhibitors (Roche/Sigma, St. Louis, MO, USA) and homogenization with a Pellet Pestle. Protein extracts were collected after sample centrifugation. Proteins were quantified with the BCA method according to the supplier’s instructions (Pierce-Thermo Scientific, Rockford, IL, USA). 50 µg of total proteins were denatured, resolved in SDS-PAGE mini-gels and electroblotted onto 0.2 µm nitrocellulose membranes (Trans-Blot Turbo Transfer Pack, Bio-Rad, Hercules, CA, USA). The membranes were blocked and incubated with specific antibodies against IGF1R (ref. 3027, Cell Signaling Technology, Beverly, MA, USA). Secondary antibody was anti-rabbit IgG-HRP (Cell Signaling Technology; Beverly, MA, USA). The immunoreactive proteins were detected with highly sensitive chemiluminescent detection reagent (ECL Prime Western Blotting Detection Reagent, Amersham; GE Healthcare Life Sciences, Pittsburgh, PA, USA). Band intensities were quantified using the Image-J Software and bands were normalized against Ponceau S staining as load control.

### Statistical analysis

Results are expressed as mean ± standard error of the mean. Normal distribution of continuous variables was tested with Shapiro-Wilk test. Comparisons were performed with unpaired t test/U-Mann Whitney or ANOVA/Kruskall-Wallis depending on the normality of the data. Statistical analyses were carried out using SPSS 21.0 (SPSS^®^ Inc. Chicago, IL, USA) and GraphPad Prism 8 (GraphPad Prism^®^, La Jolla, CA, USA). P values <0.05 were considered statistically significant.

## Results

### Efficient IGF1R depletion in perigonadal adipose tissue and liver and dimorphic sexual effects on body weight in young mice

**Figure 1:**
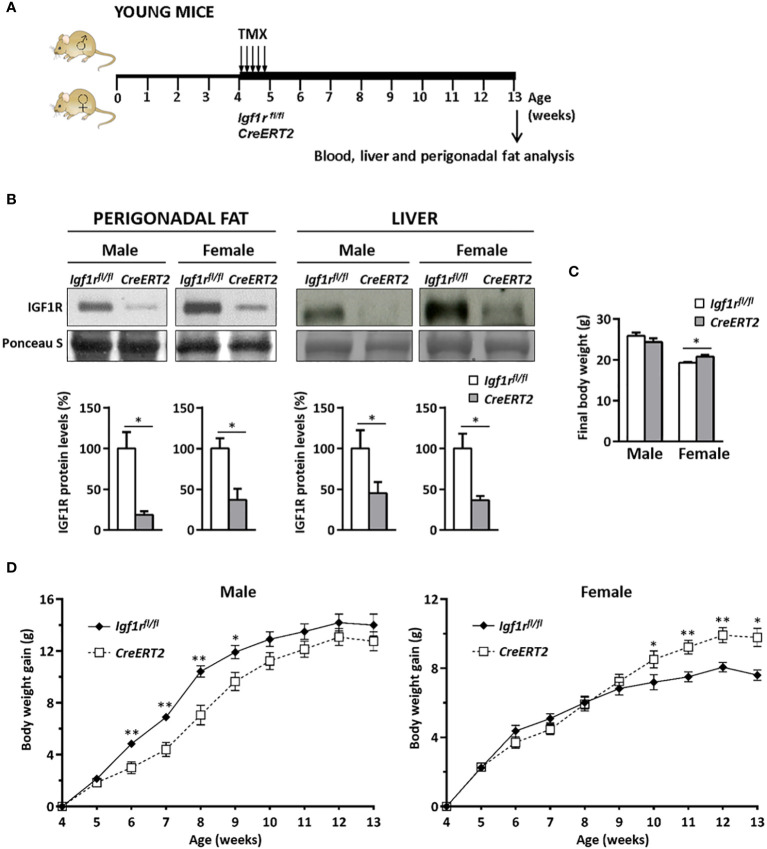
Experimental design to study the metabolic effects of IGF1R deficiency on *CreERT2* young mice, IGF1R protein levels in perigonadal fat and liver and changes in body weight gain. **(A)** Tamoxifen (TMX) was administered daily for 5 consecutive days to *Igf1r^fl/fl^* and *UBC-CreERT2; Igf1r^fl/fl^* four-weeks-old animals to generate control *Igf1r^fl/fl^* and IGF1R-deficient *CreERT2* mice. Body weight was measured weekly up to their sacrifice on week 13 for sample collection, and molecular and histological analyses. **(B)** Representative Western blots for IGF1R protein levels and graphical representation of densitometric measurements of band intensities normalized to Ponceau S staining and expressed as percentage in 13 weeks-old male (*Igf1r^fl/fl^*, n = 3; *CreERT2*, n = 3) and female (*Igf1r^fl/fl^*, n = 2; *CreERT2*, n = 3) in perigonadal fat (left) and also in liver from 13 weeks-old male (*Igf1r^fl/fl^*, n = 5; *CreERT2*, n = 5) and female (*Igf1r^fl/fl^*, n = 5; *CreERT2*, n = 4) (right). **(C)** Body weight of male (*Igf1r^fl/fl^*, n = 11; *CreERT2*, n = 14) and female (*Igf1r^fl/fl^*, n = 9, *CreERT2*, n = 10) mice at the age of 13 weeks. **(D)** Body weight gain curves of male (*Igf1r^fl/fl^*, n = 11; *CreERT2*, n = 14) and female (*Igf1r^fl/fl^*, n = 8; *CreERT2*, n = 9) mice representing the weekly mean of body weight gain on each genotype. Data are expressed as mean ± SEM. *p < 0.05, **p < 0.01 vs. *Igf1r^fl/fl^* corresponding controls.

Generation of young IGF1R-deficient mice and sample collection was performed as indicated in **Figure 1A**. IGF1R depletion in perigonadal adipose tissue and liver was confirmed by Western blot (**Figure 1B**, upper panels). A significant reduction in IGF1R protein levels was observed in both tissues of male and female *CreERT2* mice (p < 0.05). IGF1R levels in *CreERT2* perigonadal fats were reduced by 80% in males and by 60% in females respect to *Igf1r^fl/fl^* control mice, whereas in livers Reductions in IGF1R were 60% and 65%, respectively (**Figure 1B**, lower panels).

Although no significant differences were observed on final body weight of 13 weeks IGF1R-deficient young males, a significant increase in total weight was observed in *CreERT2* female mice when compared with *Igf1r^fl/fl^* controls (p < 0.05) (**Figure 1C**). In addition, different profiles of body weight gain were observed between males and females between induction of *Igf1r* gene deletion by TMX treatment and sacrifice. Whereas a significant growth deficiency was observed between six and nine weeks of age in *CreERT2* males, they finally were able to catch up normal weight. In contrast, an overweight was noticed and maintained on IGF1R-deficient females after they were 10-weeks old (**Figure 1D**).

### IGF1R deficiency in young mice alters glucose metabolism and levels of IGF1, GH, IGFBP2 and IGFBP3

**Figure 2:**
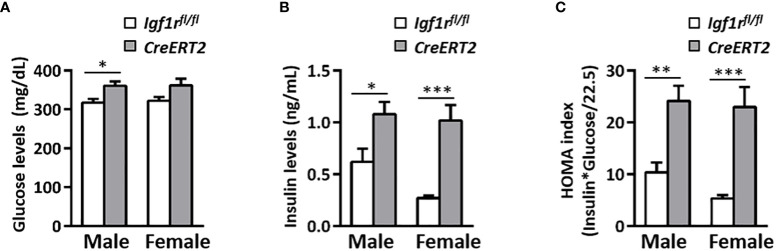
Effects of IGF1R deficiency on glucose levels and insulin resistance in young mice. **(A)** Serum glucose levels; **(B)** serum insulin levels and **(C)** the insulin resistance index (HOMA) determined in 13 weeks-old mice. Data are expressed as mean ± SEM of at least 7 animals per group (n=7-14 mice per group). *p < 0.05, **p < 0.01, ***p < 0.001 *vs Igf1r^fl/fl^* corresponding controls.

**Figure 3:**
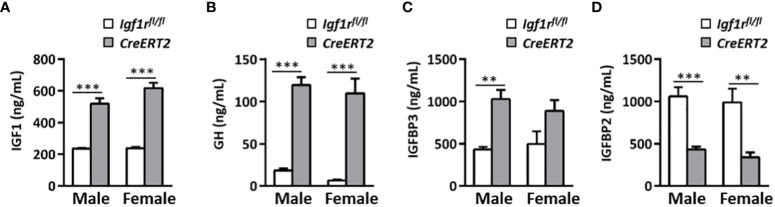
Effects of IGF1R deficiency on IGF1, GH, IGFBP3, and IGFBP2 circulating levels in young mice. Quantification of IGF1 **(A)** GH **(B)** IGFBP3 **(C)** and IGFBP2 levels **(D)** in serum of 13 weeks-old *Igf1r^fl/fl^* and *CreERT2* males and females. Data are expressed as mean ± SEM of at least 5 animals per group (n= 5-14 mice per group). **p < 0.01, ***p < 0.001 *vs*. *Igf1r^fl/fl^* corresponding controls.

Depletion of IGF1R in *CreERT2* mice was associated with a mild increase in glucose serum levels in males (p < 0.05 *vs*. *Igf1r^fl/fl^* controls) (**Figure 2A**) and with a significant elevation in insulin levels in both genders (p < 0.05 and p < 0.001 for males and females respectively) (**Figure 2B**). Accordingly, IGF1R-deficient males and females were insulin resistant based on the HOMA index when compared to their *Igf1r^fl/fl^* corresponding controls (p < 0.01-p< 0.001) (**Figure 2C**). In addition, IGF1 serum levels were found increased in both genders of *CreERT2* mice (p < 0.001), reflecting an IGF1 resistance situation (**Figure 3A**). In accordance, a significant increase in circulating levels of growth hormone (GH) was also observed in both sexes (**Figure 3B**). Since IGFBP2 and IGFBP3 regulate IGF1 bioactivity, their serum concentrations were also determined. A significant increase in IGFBP3 levels was observed in *CreERT2* males (p < 0.01). Although IGF1R-deficient females showed an elevated trend for IGFBP3, differences were not found statistically significant (**Figure 3C**). In contrast, a significant reduction in IGFBP2 levels was found in both genders of *CreERT2* mice (p < 0.001 in males; p < 0.01 in females) (**Figure 3D**). When mRNA expression levels of *Igf1*, *Igfbp3* and *Igfbp2* were analyzed in perigonadal fat and liver of young mice, their profiles followed similar trends as their respective serum levels, although only getting significance in liver (**Supplementary Figures 1A–C**).

### Impact of IGF1R deficiency on perigonadal adipose tissue and liver in young mice

**Figure 4:**
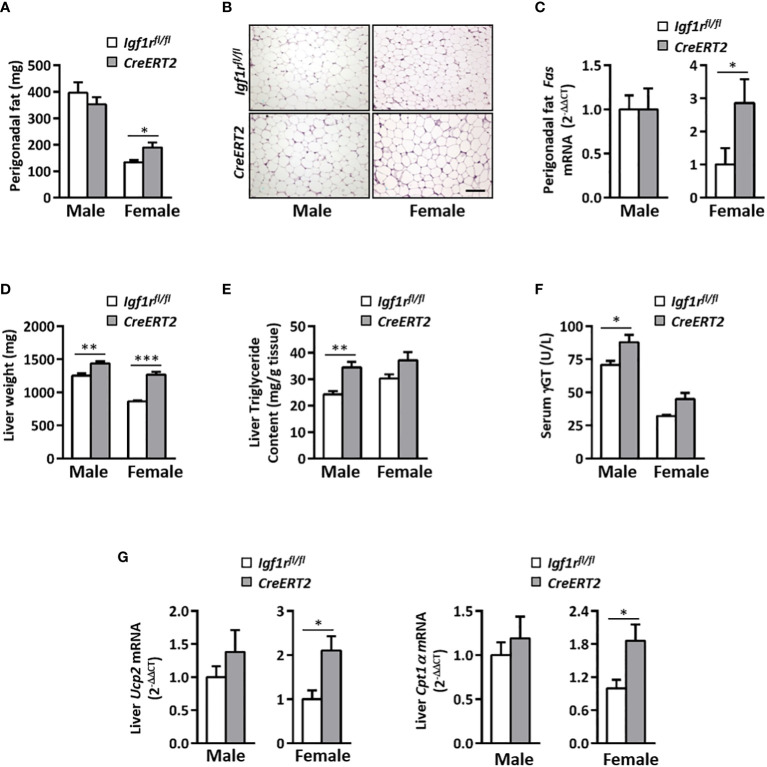
Impact of IGF1R deficiency on perigonadal adipose tissue and liver in young animals. **(A)** Effects of IGF1R depletion on perigonadal fat weight in 13 weeks-old *CreERT2* mice. **(B)** Representative H&E staining of epididymal (males) and periovarian (females) fat depots. Scale bar, 100 µm. **(C)** Perigonadal fatty acid synthase (*Fas*) mRNA levels. Gene expression is expressed as fold changes (2^-ΔΔCt^) compared to its *Igf1r^fl/fl^* corresponding controls, considered as 1. **(D)** Liver weight. **(E)** Liver triglyceride content. **(F)** Gamma-glutamyltransferase (ƴGT) circulating levels. **(G)** Liver uncoupling protein 2 (*Ucp2*) and carnitine palmitoyl-transferase 1α (*Cpt1α*) mRNA levels expressed as fold changes (2^-ΔΔCt^) and compared to their *Igf1r^fl/fl^* corresponding controls, considered as 1. Data are expressed as mean ± SEM of at least 4 animals per group (n= 4-14 mice per group). *p < 0.05, **p < 0.01 and ***p < 0.001 *vs*. *Igf1r^fl/fl^* corresponding controls.

Systemic metabolic alterations found in *CreERT2* mice lead us to analyse the impact of IGF1R depletion on perigonadal adipose tissue and liver. Although no differences were observed in total weight of epidydimal fat in males, a significant increase was observed in periovaric fat of *CreERT2* females (p < 0.05) (**Figure 4A**), in accordance to their final body weight and body weight gain (**Figures 1C, D**). A significant increase was also observed when the relative weight of periovaric fat was calculated with respect to final body weight (p < 0.05, data not shown). Despite these differences in perigonadal fat weight, their histopathological analyses did not show compelling differences (**Figure 4B**). Although adipocytes tended to be slightly bigger their divergence did not reach statistical significance (p = 0.1143, data not shown). The increased size of the perigonadal fat observed in *CreERT2* females was also accompanied by a significant increase in the mRNA levels of fatty acid synthase gene (*Fas*) (p < 0.05) (**Figure 4C**).

Liver weight was also found incremented in *CreERT2* mice of both sexes (p < 0.01 and p < 0.001 in male and female, respectively) (**Figure 4D**). Mean levels of serum γGT and liver triglycerides were significantly increased in male mutants, while no statistical differences were observed in females (**Figures 4E, F**). On the contrary, increased mRNA levels of *Ucp2* and *Cpt1α* genes, both involved in fatty acid oxidation, were observed in liver of *CreERT2* females (p < 0.05) but not in males (**Figure 4G**).

### TMX-mediated *Igf1r* deletion causes IGF1R deficiency in perigonadal fat and liver and reduces body weight in middle-aged mice

**Figure 5:**
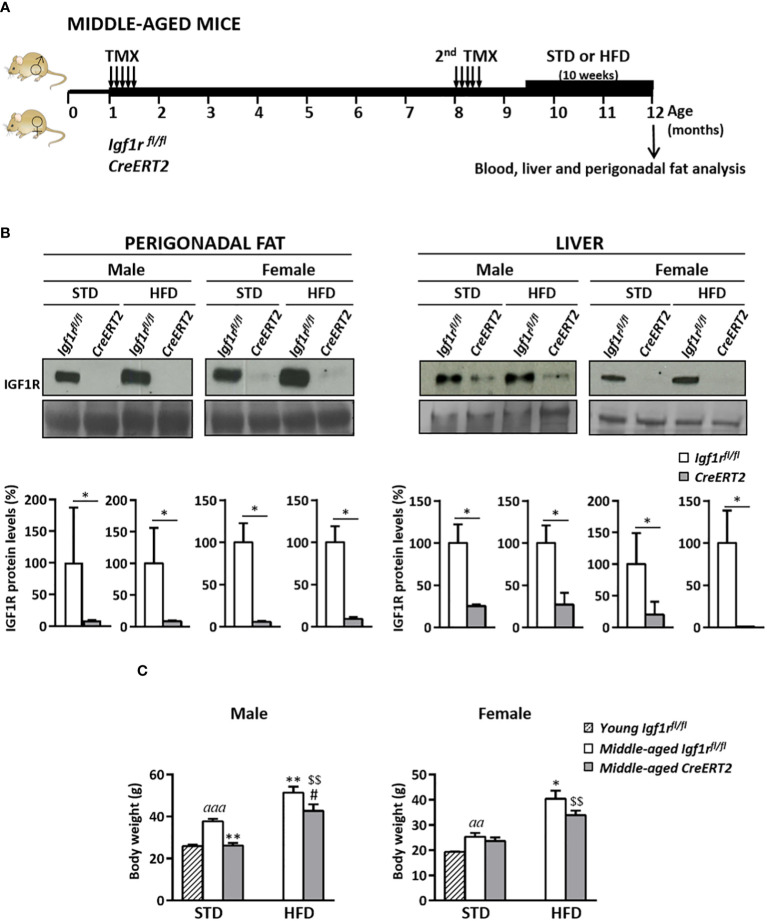
Experimental design to study the metabolic effects of IGF1R deficiency in middle-aged mice, IGF1R protein levels and changes in body weight in middle-aged IGF1R-deficient mice either on normal feeding or after a high-fat diet challenge. **(A)** TMX was administered daily for 5 consecutive days to *Igf1r^fl/fl^* and *UBC-CreERT2; Igf1r^fl/fl^* in two sessions, the first to four-weeks-old and the second to eight-months-old animals, to generate control *Igf1r^fl/fl^* and IGF1R-deficient (*CreERT2)* mice. Body weight was measured weekly up to their sacrifice on month 12. Nine and a half month-old *Igf1r^fl/fl^* and *CreERT2* mice were subdivided into two different diet groups: one kept on normal standard diet (STD), and the other changed to a high-fat diet (HFD) feeding during the last 10 weeks before their sacrifice. **(B)** Representative Western blots for IGF1R protein levels and graphical representation of densitometric measurements of band intensities normalized to Ponceau staining in perigonadal fat and liver of one year-old middle-aged male and female IGF1R-deficient mice (*CreERT2*) with respect to controls (*Igf1r^fl/fl^*), either fed with standard (STD) or challenged with HFD for the last 10 weeks (n= 2-6 mice per group). **(C)** Final body weight of middle-aged *Igf1r^fl/fl^* and *CreERT2* mice on STD and HFD, compared to young *Igf1r^fl/fl^* control (generated as mentioned in **Figure 1A**). Body weight data are expressed as mean ± SEM of at least 4 animals per group (n= 4-11 mice per group). *^aa^p* < 0.01 and *^aaa^p* < 0.001 *vs*. *Igf1r^fl/fl^* young mice; *p < 0.05, **p < 0.01 *vs*. *Igf1r^fl/f^* middle-aged STD animals; ^#^p < 0.05 *vs. Igf1r^fl/fl^* middle-aged HDF mice, and ^$$^ *p* < 0.01 *vs.* middle-aged STD *CreERT2* mice.

In order to study the effect of aging and a high-fat diet (HDF) on systemic metabolism, as well as on liver and perigonadal fat in middle-aged IGF1R-deficient mice, *CreERT2* mice were generated and analysed at the age of twelve months, either with or without a HFD challenge for 10 weeks, following the protocol shown in **Figure 5A**. Efficient depletion of IGF1R protein levels in perigonadal adipose tissue and liver of *CreERT2* middle-aged mice was confirmed by Western-blotting (**Figure 5B**, upper panels). Reductions in IGF1R levels of 90% in perigonadal fat and higher than 80% in liver of *CreERT2* mice of both sexes on standard-diet (STD) and HFD were determined by densitometric quantification (**Figure 5B** lower panels). IGF1R deficiency efficiently counteracted the significant increase in body weight induced by aging and by the ingestion of a HFD in both sexes, although without statistical significance in *CreERT2* females on STD (**Figure 5C**).

### Effects of IGF1R deficiency on glucose metabolism of middle-aged mice

**Figure 6:**
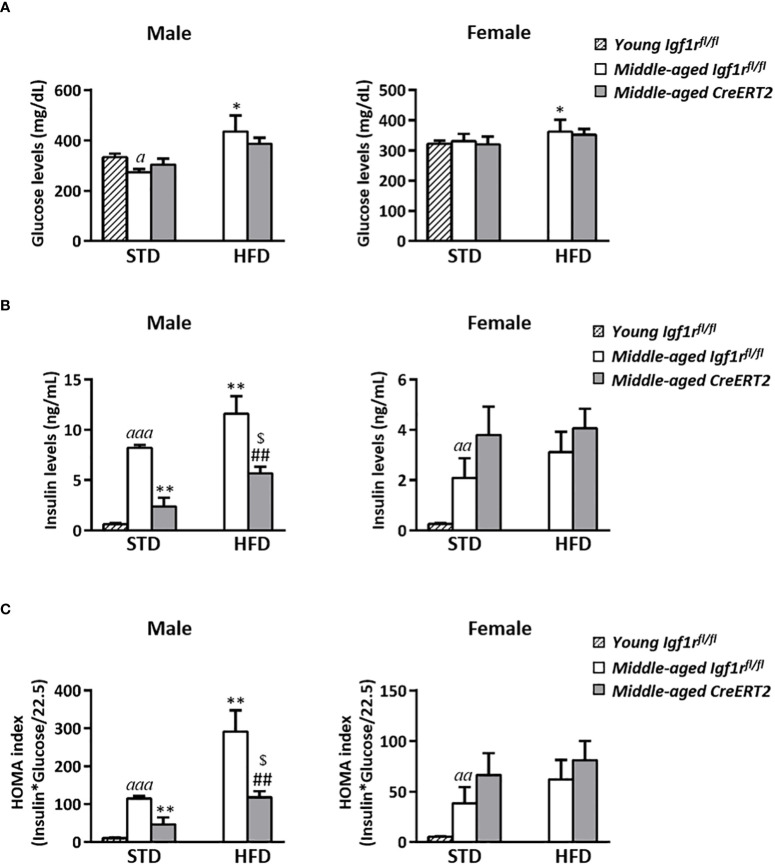
Glucose homeostasis in middle-aged male IGF1R-deficient mice. Serum levels of glucose **(A)** and insulin **(B)**; and insulin resistance index (HOMA) **(C)** in *CreERT2* IGF1R-deficient middle-aged male and female mice compared to their *Igf1r^fl/fl^* counterparts and to young (13 weeks-old) *Igf1r^fl/fl^* on STD. Data are expressed as mean ± SEM of at least 4 animals per group (n= 4-11 mice per group). *^a^p* < 0.05, ^aa^ *p* < 0.01 and *^aaa^p* < 0.001 *vs*. young *Igf1r^fl/fl^* STD mice. *p < 0.05, **p < 0.01 *vs*. middle-aged *Igf1r^fl/f^* STD animals; ^##^p < 0.01 *vs.* middle-aged *Igf1r^fl/fl^* HFD mice; and ^$^ *p* < 0.05 *vs.* middle-aged *CreERT2* STD animals.

Middle-aged control male mice on STD presented slightly lower glucose levels than their young *Igf1r^fl/fl^* counterparts, however, such difference was not observed in *CreERT2* mice. The ingestion of a HFD by middle-aged *Igf1r^fl/fl^* male mice significantly increased glucose levels respect to *Igf1r^fl/fl^* STD animals (p < 0.05) (**Figure 6A**, left). In females, apart from a significant augment in glucose levels of *Igf1r^fl/fl^* due to the ingestion of the HFD, no other differences were observed (**Figure 6A**, right). A considerable significant increase in insulin levels was observed in middle-aged *Igf1r^fl/fl^* males on STD that was further incremented upon HFD. IGF1R deficiency was able to counteract such increase in males under both types of diets (STD or HFD) (p < 0.01) (**Figure 6B**, left). In females, insulin levels increased with ageing, but no significant changes were induced by HFD diet or IGF1R depletion (**Figure 6B**, right). In accordance, the HOMA index followed insulin levels profiles (**Figure 6C**).

### Aging and IGF1R deficiency alters IGF1, GH and IGFBPs serum levels on middle-aged mice upon STD and HFD

**Figure 7:**
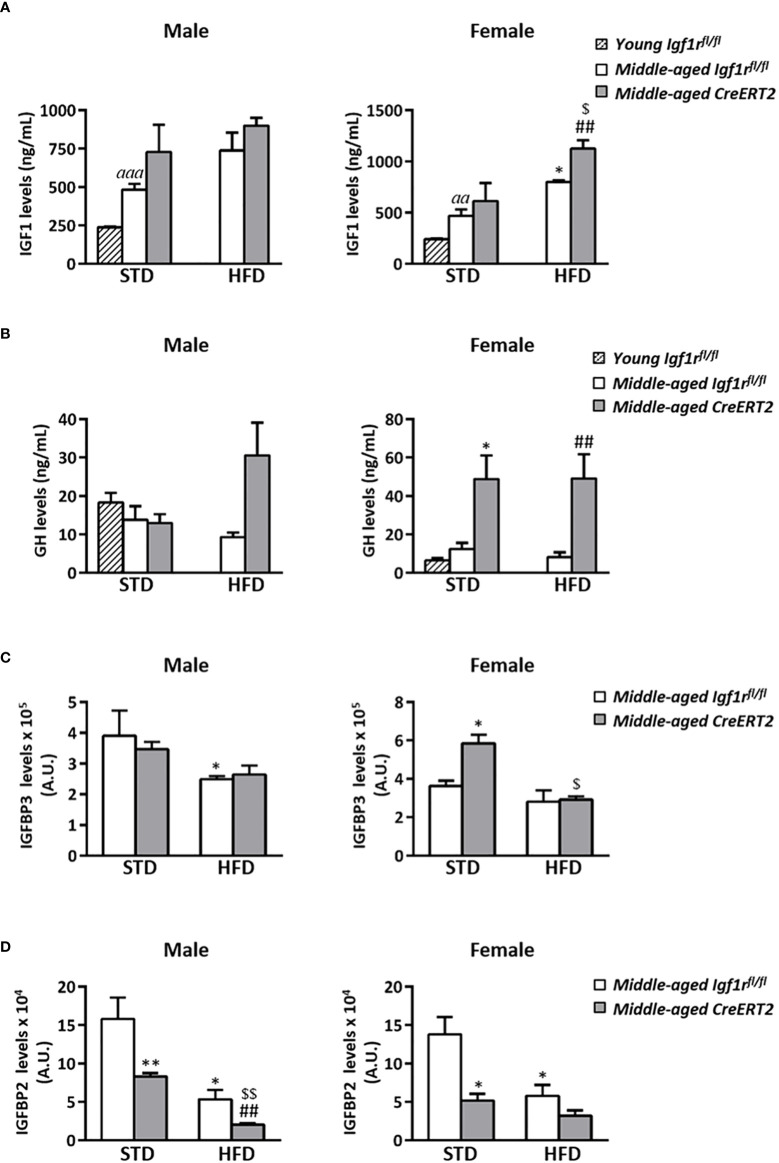
Impact of IGF1R deficiency on IGF1, GH, IGFBP3 and IGFBP2 serum levels of middle-aged mice. Quantification of circulating levels of IGF1 **(A)** GH **(B)** IGBP3 **(C)** and IGFBP2 **(D)** in *CreERT2* male and female middle-aged mice on STD and HFD compared to *Igf1r^fl/fl^* counterparts and to young control (*Igf1r^fl/fl^*) male and females on STD. Data are expressed as mean ± SEM of at least 2 animals per group (n= 2-11 mice per group). ^aa^ *p* < 0.01 and *^aaa^p* < 0.001 *vs*. young *Igf1r^fl/fl^* mice. *p < 0.05, **p < 0.01 *vs*. middle-aged *Igf1r^fl/fl^* STD animals; ^##^p < 0.01 *vs.* middle-aged *Igf1r^fl/fl^* HFD mice, and ^$^ *p* < 0.05, ^$$^ *p* < 0.01 *vs.* middle-aged *CreERT2* STD animals.

Aging induced a significant increase in IGF1 levels in both males and females (p < 0.001 and p < 0.01 respectively). Although no significant effects were observed after the ingestion of a HFD or due to the mutation in middle-aged male mice, a significant increase in IGF1 levels was observed on female mice fed with a HFD (p < 0.05). Interestingly, this increase was further pronounced by IGF1R deficiency (**Figure 7A** right). No significant effects were observed on GH circulating levels in male mice, whereas a significant increase was observed in IGF1R-deficient females under both types of diets (STD or HFD) (**Figure 7B** right). IGFBP3 serum levels were diminished in middle-aged *Igf1r^fl/fl^* control males fed with a HFD (p < 0.05), but were not affected by IGF1R deficiency (**Figure 7C**, left). On the contrary, IGFBP3 serum levels in females were not affected by diet in *Igf1r^fl/fl^* controls, but were increased in *CreERT2* females on STD. Interestingly, IGF1R deficiency counteracted this increase in females fed with HFD (**Figure 7C**, right). Finally, IGF1R deficiency was accompanied by a significant reduction in IGFBP2 in middle-aged male and female mice fed with either STD or HFD (**Figure 7D**), similarly to what was observed in young mice (**Figure 3D**). mRNA levels of *Igf1*, *Igfbp3* and *Igfbp2* locally analyzed in perigonadal fat and liver of middle-aged mice showed a similar trend to those of their serum levels (**Supplementary Figure 2**).

### IGF1R deficiency couteracts perigonadal adipose tissue deposition and inflammation in middle-aged male mice

**Figure 8:**
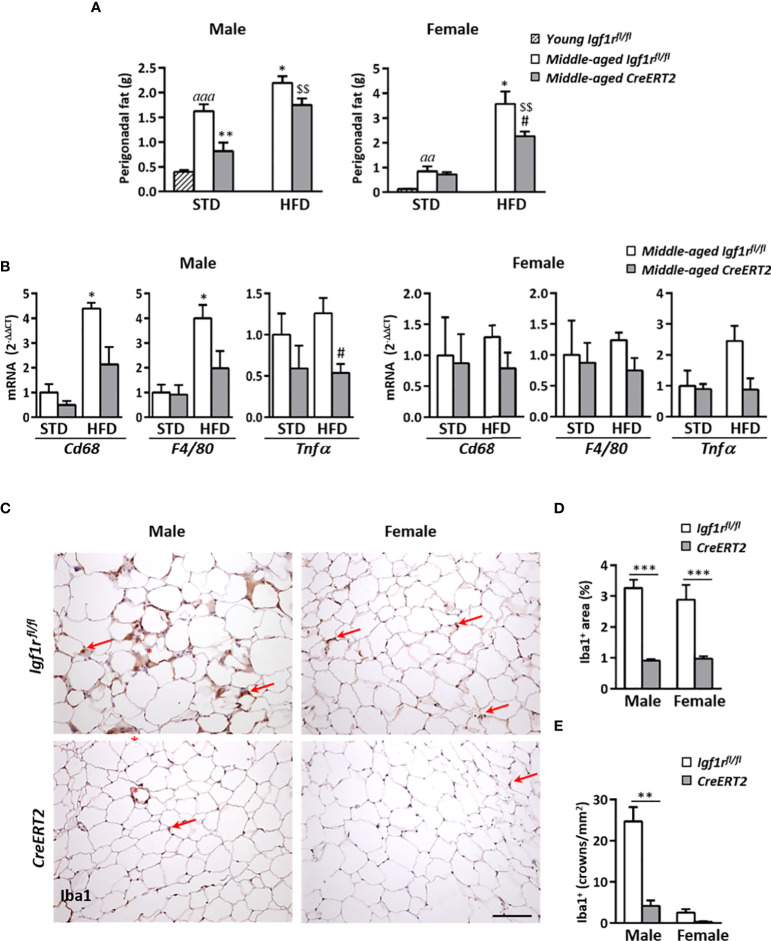
Effects of IGF1R deficiency on perigonadal adipose tissue content and inflammation in middle-aged mice on HFD diet. **(A)** Effects of IGF1R depletion on perigonadal fat weight in male and female mice fed with either STD or HFD compared to young control (*Igf1r^fl/fl^*) male and females on STD (n= 4-10 mice per group). **(B)** mRNA levels of inflammatory markers *Cd68*, *F4/80* and *Tnfα* mRNA levels in midlle-aged mice fed either with STD or HFD. Gene expression is expressed as fold changes (2^-ΔΔCt^) compared to its *Igf1r^fl/fl^* corresponding control group. Data are expressed as mean ± SEM of at least 4 animals per group. **(C)** Representative immunohistochemical stainings of perigonadal fat tissue for the specific macrophage marker Iba1 (red arrows) in middle-aged mice fed with HFD. Crown-like structures, composed of macrophages surrounding adipocytes, are indicated with red asterisks. Scale bar: 100 µm. **(D)** Graphical representation of Iba^+^ stained area quantification (percentage) shown in **(C)**. **(E)** Quantification of crown-like Iba1^+^ structures (density). Data in **(D, E)** are expressed as mean ± SEM of 2-10 animals per group (data obtained per animal analyzed in duplicated sections). ^aa^ *p* < 0.01 and *^aaa^p* < 0.001 *vs*. young *Igf1r^fl/fl^* mice; *p < 0.05 and **p < 0.01 *vs*. middle-aged *Igf1r^fl/fl^* STD animals; ^#^p < 0.05 *vs.* middle-aged *Igf1r^fl/fl^* HFD middle-aged mice; and ^$$^ *p* < 0.01 *vs.* middle-aged *CreERT2* STD animals in **(A, B)** **p < 0.01 and ***p < 0.001 *vs*. corresponding *Igf1r^fl/fl^* mice, in **(D, E)**.

Due to the effects on body weight reduction caused by IGF1R deficiency on middle-aged mice, liver and perigonadal fat weights were also evaluated. Middle-aged *Igf1r^fl/fl^* mice on STD showed higher liver weight than young mice, as also did *Igf1r^fl/fl^* males on HFD (p < 0.01). However, no significant effects were observed due to IGF1R deficiency neither in males nor in females (**Supplementary Figure 3A**). Similar profiles were found for hepatic triglyceride content (**Supplementary Figure 3B**). Aging was accompanied by a significant increase in perigonadal fat in *Igf1r^fl/fl^* middle-aged mice of both sexes respect to young animals, being this increase more pronounced in males (**Figure 8A**). Interestingly, IGF1R deficiency was able to counteract this increase in males fed with both types of diet (**Figure 8A** left), but only in females on HFD (**Figure 8A** right).

As obesity is associated with adipose tissue inflammation, mRNA levels of genes involved in macrophage infiltration (*Cd68* and *F4/80*) and proinflammatory action (*Tnfα*) were also evaluated in perigonadal fat tissue. HFD feeding led to a significant increase in mRNA levels of *Cd68* and *F4/80* genes in *Igf1r^fl/fl^* control males whereas *CreERT2* males showed a tendency to impair such increase. In addition, expression of *Tnfα* was significantly reduced in *CreERT2* males on HFD respect to *Igf1r^fl/fl^* controls on the same diet (**Figure 8B**, left). However, there were not significant changes in expression of any of these genes in females of any condition (**Figure 8B**, right). Immunostaining for Iba1, a macrophage marker used to identify presence of these cells in perigonadal fat of HFD mice, confirmed these results. As shown in **Figure 8C**, total Iba1^+^ immunostained area and presence of macrophage crown-like structures in perigonadal fat tissue of middle-aged *Igf1r^fl/fl^* males was highly reduced after induction of IGF1R depletion (**Figure 8C**, left panels). The same trend was observed in females, although the presence of crown-like structures was highly reduced in both genotypes (**Figure 8C**, right panels). Quantification of Iba1^+^ immmunostained area (**Figure 8D**), and presence of macrophage crowns-like structures (**Figure 8E**), confirmed the anti-inflammatory effect of IGF1R deficiency in perigonadal adipose tissue of middle-aged male mice.

## Discussion

Here we report how a generalized IGF1R deficiency in mice alters the systemic metabolism homeostasis in a sex and age dependent manner with differential impacts on liver and perigonadal adipose tissue of young and HFD-challenged middle-aged mice. We show that whereas young mice were insulin-resistant, with increased IGF1 and GH levels as well as altered IGFBPs in serum, IGF1R-deficient middle-aged mice reverted such alterations, even when they were on a HFD. In addition, high insulin levels, adiposity and adipose-associated inflammation levels induced by HFD were also counteracted by IGF1R depletion in middle-aged males.

TMX-mediated IGF1R deficiency induced in prepuberal *UBC-CreERT2; Igf1r^fl/fl^* mice was found to be highly effective at the protein level in both perigonadal adipose tissue and liver (> 60%), two insulin target organs with important roles in metabolism homeostasis. Accordingly, efficient depletion in IGF1R mRNA and protein levels was previously proved in different tissues of these *CreERT2* mutant mice (25, 35–37). Interestingly, *CreERT2* middle-aged mice (1-year old) showed even a higher reduction in IGF1R protein expression in these tissues (> 90% in perigonadal fat and > 75-80% in liver), most probably due to the second dose of TMX administered at the age of 8 months.

*CreERT2* young mice showed increased IGF1, GH and IGFBP3 circulating levels while IGFBP2 levels were found reduced. However, with the exception of IGFBP2, these effects were not so clear in middle-aged *CreERT2* mice. In accordance with the results observed in young animals, mice with compromised IGF1R signaling were previously reported to show high serum IGF1, GH and IGFBP3 levels (22, 38). It is known that circulating levels of IGFBP3 are elevated by IGF1 (39). However, high levels of GH were unexpected, since GH levels are usually downregulated by IGF1 *via* somatostatin negative feedback in the pituitary gland (10), suggesting a partial resistance to IGF1 actions. Interestingly, high levels of IGF1 have also been described in patients with heterozygous or hemizygous deletions resulting in loss of function of *IGF1R* (8, 40–42). Increased IGFBP3 and reduced IGFBP2 levels were also reported in patients with *IGF1R* mutations which was interpreted as a supraphysiological secretion of GH (42). Since *CreERT2* mice reflect systemic levels of IGF1 and related proteins found in patients with mutations in the *Igf1r* gene, these mice appear to be useful as a model to understand the human pathophysiology of this condition.

We found that young but not middle-aged IGF1R-deficient mice (both males and females) were insulin resistant. IGF1 is involved in nutrient sensing responses to insulin. In fact, IGF-1 may indirectly modulate carbohydrate metabolism through GH suppression and enhancement of insulin action (10). Disruption of IGF1R signaling in mice has led to some degree of glucose intolerance and insulin resistance in most models examined (43) including ours. Thus, transgenic mice expressing a kinase-deficient IGF1R β-subunit with reduced signal transduction in both IGF1R and IGF1R/InsR hybrid receptors developed diabetes early on life (14, 44). Also, male mice carrying a genetic mutation that lack one of the *Igf1r* alleles (*Igf1r* ^+/−^) were glucose intolerant and both males and females developed insulin resistance as they age (24). Other models of IGF1R or InsR/IGFIR double-knockout in brown adipose tissue also revealed insulin resistance (17, 45). Furthermore, *UBIKOR* IGF1R-deficient mice that were generated using the same genetic strategy than our *CreERT2* mice also developed a mild glucose intolerance and insulin resistance (22). However, it is not fully understood whether insulin resistance in these animal models is due to GH overproduction, IGF modulatory actions (46), or other IGF-dependent events or network components. In this regard, previous studies have shown that overexpression of IGFBP3 is associated with impaired glucose tolerance (47–49). The increased IGFBP3 levels in young *CreERT2* mice, along with its increased expression in liver and perigonadal adipose tissue, may suggest a potential involvement of this binding protein in inducing insulin resistance.

Impact of IGF1R deficiency in body weight gain was sex dimorphic in young mice. Whereas *CreERT2* males were retarded in body weight gaining shortly after the post-weaning TMX treatment with a final catch up in weight during the last month, IGF1R-deficient females showed a higher weight gaining during the last month; a sexual dimorphism that could be due to different compensatory mechanisms between males and females. These results differ from those reported by Francois et al. (2017) in *UBIKOR* mice, where they found similar growth arrest in both sexes of mice (22). Of note, IGF1R deficiency in young mice increased perigonadal fat deposition only in females. This higher fat content could contribute to increased body weight found in this sex. Interestingly, the high *Fas* mRNA levels observed in perigonadal fat of *CreERT2* females could be responsible for increased lipogenesis observed in this tissue (50).

Young *CreERT2* mice presented a significant increase of liver weight in both genders. Interestingly, local expression of mRNA levels of IGF1 and IGFBP3 followed the same trend. Since liver is a rich source of these proteins in circulation, these results suggest that their expression in liver could be somehow counter-regulated by IGF1R. In addition, the increase of liver weight could be explained, at least in part, by the increased liver triglyceride content observed, particularly in males, although without evident histopathological alterations. Increased ɤGT levels corroborate such findings. In accordance, *UBIKOR* mice that also display bigger livers neither showed histoanatomical alterations in this organ (22). Tendency to hepatic accumulation of triglycerides in females could be due to the significant increase in mRNA levels of *Cpt1α* and *Ucp2* genes. CPT1α is responsible for the transport of fatty acids into the mitochondria, and *Ucp2* is a key protein for the mitochondrial oxidation of fatty acids. Thus, our findings could suggest a compensatory pathway for IGF1R deficiency, trying to counteract the increased accumulation of triglycerides in the liver that was only efficient in females. Phenotypical discrepancies between *CreERT2* mice described here, and *UBIKOR* mice reported by Francois et al. (2017) (22), could be due to mouse differences in genetic background and mutations design. In addition, it could also depend on the experimental set ups used to induce *Igf1r* gene deletion. Thus, whereas TMX-mediated gene deletion in *CreERT2* mice is induced in prepuberal mice (4 weeks old), in UBIKOR mice was performed in 3 months-old adult mice, where scarce receptors can be found in liver and adipose tissue (22, 51).

Results shown here further demonstrate that metabolic consequences of IGF1R signaling disruption change with aging. In this sense, middle-aged *CreERT2* mice did no show significant differences on liver weight or triglyceride accumulation neither in males nor in females, in contrast to what was observed in young mice. Our study showed that aging caused an increase in serum IGF1 without altered GH levels. These results contrast with previous studies reporting that circulating levels of GH and, consequently, hepatic production of IGF1 significantly decline with age in both humans and rodents (52–54). These discrepancies could be explained by the fact that our “middle-aged mice” could not be strictly considered as “aged” (22-26 months old) mice, in which altered dynamics in IGF1/GH was described. Interestingly, IGF1R deficiency in middle-aged females was accompanied by a significant increase in IGF1, GH and IGFBP-3 levels, suggesting a possible IGF1 resistance effect, whereas these differences were not observed in males. Thus, a clear sex dimorphic pattern was observed in middle-aged mutants affecting IGF1/GH network that was not present in young mice.

Aging *per se* is an independent causative factor contributing to the development of metabolic syndrome (55–57) and insulin resistance plays a principal role in its initialisation and perpetuation (58). In this context, our study demonstrates that aging is associated with increased insulin resistance in both genders of control mice. In addition, the ingestion of a HFD for 10 weeks exacerbates such situation in males but not in females, suggesting that middle-aged females display protective actions against the negative effects of a HFD. Since estrogens are recognized key regulators of energy balance and glucose homeostasis (59–61), they could protect females but not males on HFD to develop insulin resistance, independently of the IGF1R signaling activity. Interestingly, IGF1R deficiency in males but not in females resulted in resistance to aging- or HFD-induced insulin resistance and increased adiposity, in opposition to previous studies (15, 24, 51). Our findings agree with the study from Boucher et al. (2012) which shows that fat specific IGF1R and insulin receptor double knock out mice were resistant to age-associated and diet-induced obesity and glucose intolerance (15). Overall, our findings suggest that targeting IGF1R may be a promising strategy to counteract the deleterious effects of aging and/or HFD on adiposity and glucose metabolism, at least in middle-aged males. In follow-up studies, the timing of IGF1R targeting should be assessed in order to address the question if IGF1R targeting has acute or long-term effects (e.g. metabolic programming) on metabolism. Notably, overexpression of IGFBP2 in mice also revealed positive effects on glucose metabolism that where linked with obesity and were identified only at older ages (62).

It is well-known that adiposity increases recruitment of macrophages in adipose tissue and that adipose tissue inflammation underlies the etiology of obesity and its two major comorbidities, insulin resistance and type 2 diabetes (reviewed by Dahik et al., 2020) (63). *Igf1* is expressed by multiple cell types in adipose tissue, including adipocytes and macrophages. Both adipocyte- and macrophage-derived IGF1 contribute to local tissue homeostasis and adaptation to metabolic stress (64). Thus, the increased macrophage presence in adipose tissue in obesity could explain, at least in part, the significant increase observed in IGF1 levels after the ingestion of a HFD. We demonstrate that a generalized disruption of IGF1R signalling has a protective action against aging and HFD-induced obesity, specifically by reducing adipose tissue inflammation. In this regard, ablation of the macrophage IGF1-IGF1R axis inhibits the NLRP3 inflammasome, a protein complex responsible for the activation of inflammatory responses, which indicates that IGF1R plays an important role in initiation of the inflammatory process (65). Accordingly, IGF1R deficiency was reported to confer resistance to bleomicyn-mediated lung injury, house dust mite-induced allergy and lung tumor microenvironment pulmonary inflammatory response (35–37). Altogether, these findings support IGF1R as an important player in murine tissue inflammation, suggesting that targeting IGF1R may counteract the inflammatory component of multiple inflammatory diseases.

## Conclusions

Our study has demonstrated that postnatal generalized IGF1R deficiency mimics metabolic alterations observed in patients carrying mutations in *IGF1R*, such as insulin resistance, changes in serum IGF and IGFBPs and fatty liver. Therefore, this mouse model could be a useful tool to better understand the mechanisms underlying the pathophysiology of this mutation in humans. However, these findings contrast with those observed in IGF1R-deficient middle-aged mice, where these effects where mostly counteracted, suggesting potential compensatory signaling effects as mice age. In addition, our results show that IGF1R deficiency prevents the negative effects induced by aging and HFD on adiposity, inflammation and glucose metabolism, specifically in middle-aged male mice, indicating a clear metabolic sexual dimorphism dependent on IGF1R signaling. Notably, our findings contribute to understand the importance of IGF1R in systemic metabolism, adiposity and inflammation.

## Data availability statement

The raw data supporting the conclusions of this article will be made available by the authors, without undue reservation.

## Ethics statement

The animal study was reviewed and approved by Institutional Animal Care & Use Committee (IACUC) from the Center for Biomedical Research of La Rioja (CIBIR), Spain (ref 03/12).

## Author contributions

PPM, JAO and JGP designed the study. IL, MI, ERF, RT, SPH, EAA, LC, CW and AH performed the experiments and collected the data. PPM, IL, MI and AH analysed the data. PPM, JAO and JGP interpreted the data. PPM wrote the paper. All authors reviewed and/or edited the manuscript. All authors contributed to the article and approved the submitted version.

## Funding

This work was supported by Fundación Rioja Salud (La Rioja Government) to PPM and by the Spanish MCIN/AEI/10.13039/501100011033 (Project PGC2018-097397-B-I00) and the Fundacioín Rioja Salud Project 6.FRS-ABC.006 to JGP, a co-founded by the European Regional Development and European Social Funds (ERDF/ESF).

## Acknowledgments

We would like to thank Dr. MJ Villanueva-Millán for her help with experiments.

## Conflict of interest

The authors declare that the research was conducted in the absence of any commercial or financial relationships that could be construed as a potential conflict of interest.

## Publisher’s note

All claims expressed in this article are solely those of the authors and do not necessarily represent those of their affiliated organizations, or those of the publisher, the editors and the reviewers. Any product that may be evaluated in this article, or claim that may be made by its manufacturer, is not guaranteed or endorsed by the publisher.

## Supplementary material

Supplementary Figure 1 Effects of IGF1R depletion on perigonadal and hepatic mRNA expression levels of IGF system genes in young mice. qPCR was performed to determine *Igf1* **(A)**, *Igfbp3* **(B)** and *Igfbp2* **(C)** mRNA levels on young (13 week-old) males and females, comparing IGF1R-deficient (*CreERT2*) respect to control (*Igf1r^fl/fl^*) mice. Values are means ± SEM of 4-7 animals per group. Gene expression is expressed as fold changes (2^-ΔΔCt^) compared to its corresponding *Igf1r^fl/fl^* mice (control group), which was considered as 1. *p < 0.05, **p < 0.01 *vs*. corresponding controls.

Supplementary Figure 2 Effects of IGF1R deletion on perigonadal and hepatic mRNA expression levels of IGF system genes in midde-aged mice on STD and HFD. qPCR was performed to determine **(A)** *Igf1*, **(B)** *Igfbp3* and **(C)** *Igfbp2* mRNA levels on middle-aged males and females, comparing IGF1R-deficient *CreERT2* respect to control *Igf1r^fl/fl^* mice. Values are means ± SEM of 3-8 animals per group. Gene expression is expressed as fold changes (2^-ΔΔCt^) compared to its corresponding *Igf1r^fl/fl^* mice (control group), which was considered as 1. ^#^p < 0.05 *vs. Igf1r^fl/fl^* mice fed with a HFD and ^$^ *p* < 0.05 *vs. CreERT2* animals fed with STD.

Supplementary Figure 3 Impact of IGF1R deletion on liver in middle-aged mice on STD and HFD diet. **(A)** Effects of IGF1R deletion on liver weight; and **(B)** on liver triglyceride content in males and females fed with either a STD or HFD and compared to young (13 weeks-old) control (*Igf1r^fl/fl^*) male and females on STD. Values are means ± SEM of 4-11 animals per group. *^aa^p* < 0.01 and *^aaa^p* < 0.001 *vs*. young *Igf1r^fl/fl^* mice. *p < 0.05, **p < 0.01 *vs*. middle-aged *Igf1r^fl/fl^* STD animals.

Supplementary Table 1 Primers sequences used for real-time PCR.
