# Supplementary figures and images for: IGF1R is a mediator of sex-specific metabolism in mice: Effects of age and high-fat diet
Source: Front Endocrinol (Lausanne). 2022 Oct 20;13:1033208. doi: 10.3389/fendo.2022.1033208 (PMC9638844; doi:10.3389/fendo.2022.1033208)

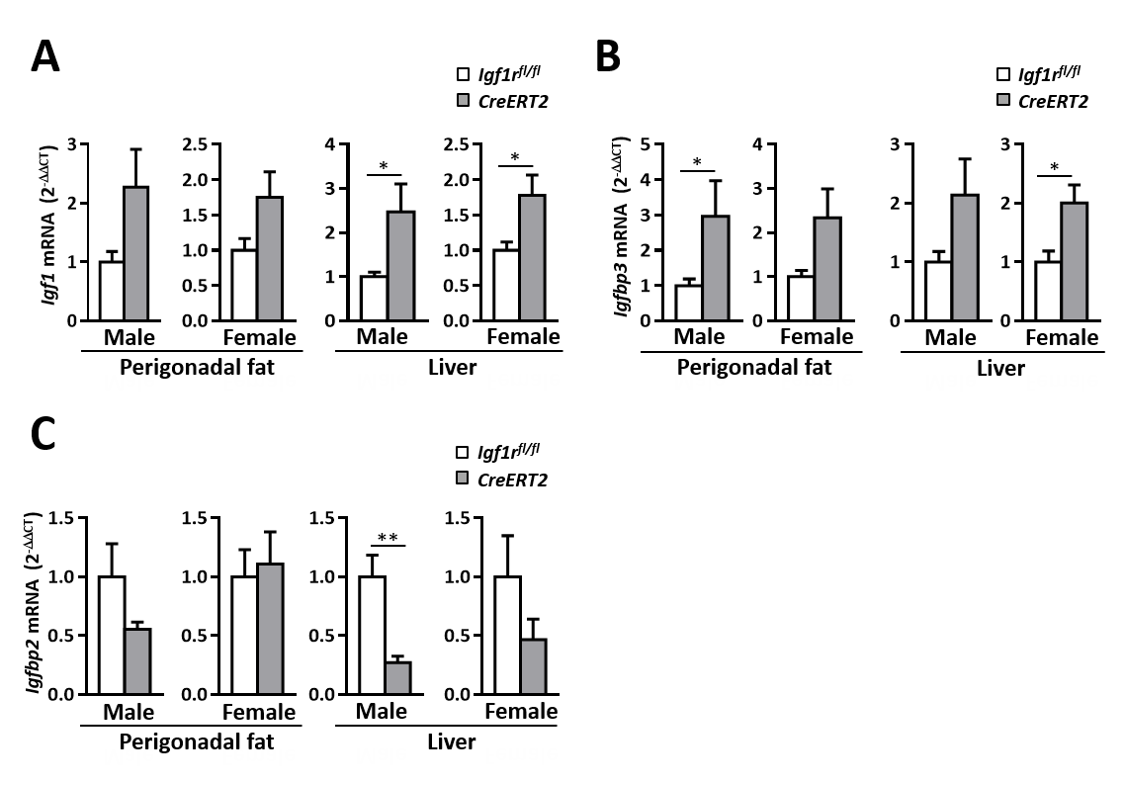

Supplement: Supplementary Figure 1 — Effects of IGF1R depletion on perigonadal and hepatic mRNA expression levels of IGF system genes in young mice. qPCR was performed to determine Igf1 (A), Igfbp3 (B) and Igfbp2 (C) mRNA levels on young (13 week-old) males and females, comparing IGF1R-deficient (CreERT2) respect to control (Igf1rfl/fl ) mice. Values are means ± SEM of 4-7 animals per group. Gene expression is expressed as fold changes (2-ΔΔCt) compared to its corresponding Igf1rfl/fl mice (control group), which was considered as 1. *p < 0.05, **p < 0.01 vs. corresponding controls. [file Image_1.tif]

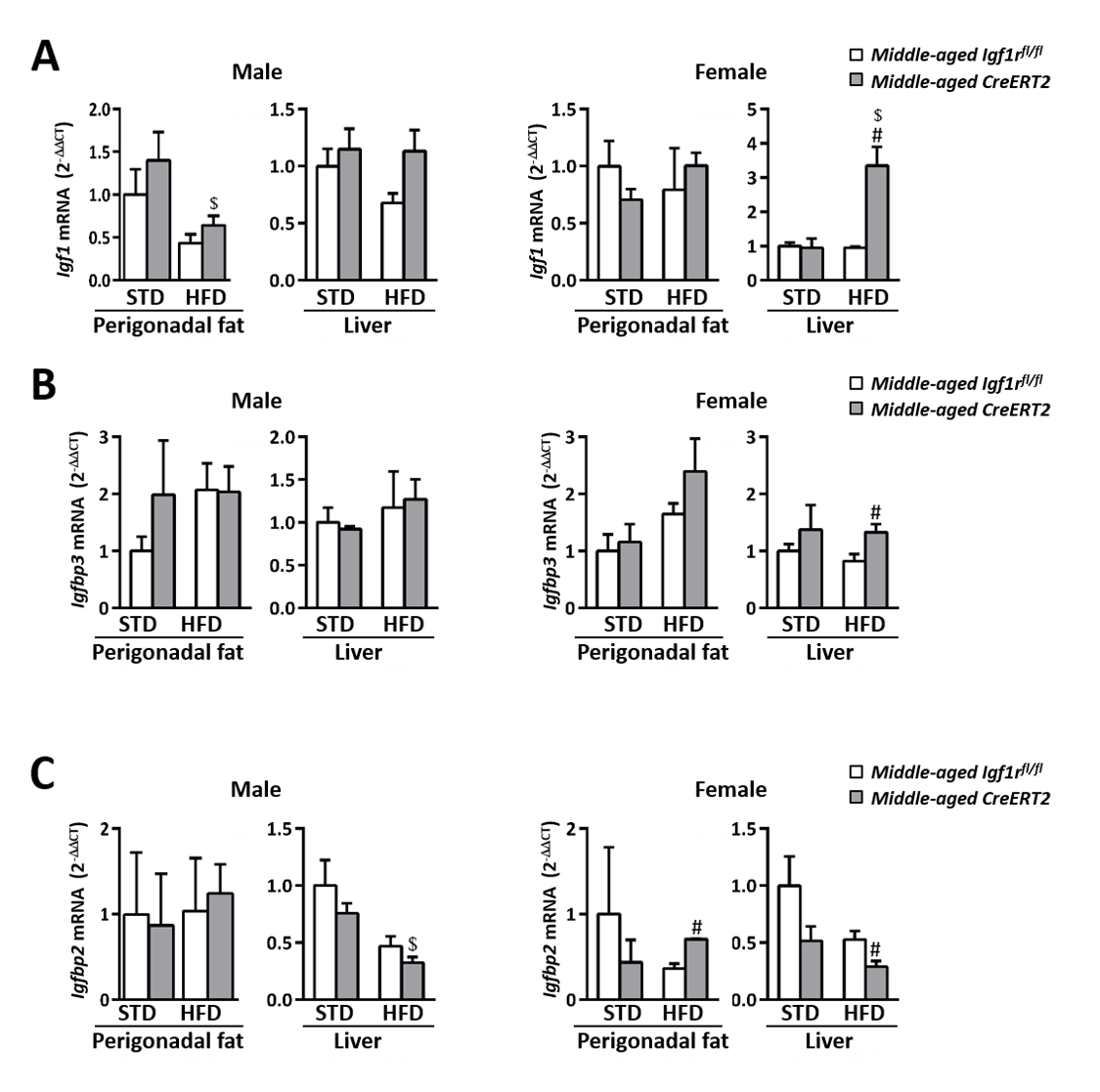

Supplement: Supplementary Figure 2 — Effects of IGF1R deletion on perigonadal and hepatic mRNA expression levels of IGF system genes in midde-aged mice on STD and HFD. qPCR was performed to determine (A) Igf1, (B) Igfbp3 and (C) Igfbp2 mRNA levels on middle-aged males and females, comparing IGF1R-deficient CreERT2 respect to control Igf1rfl/fl mice. Values are means ± SEM of 3-8 animals per group. Gene expression is expressed as fold changes (2-ΔΔCt) compared to its corresponding Igf1rfl/fl mice (control group), which was considered as 1. #p < 0.05 vs. Igf1rfl/fl mice fed with a HFD and $ p < 0.05 vs. CreERT2 animals fed with STD. [file Image_2.tif]

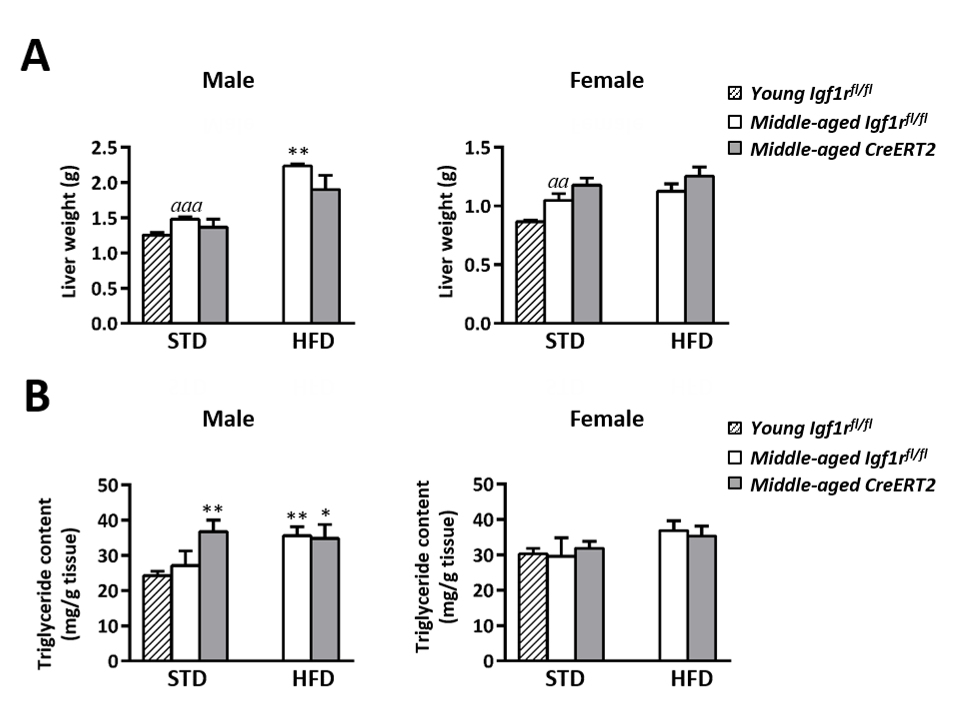

Supplement: Supplementary Figure 3 — Impact of IGF1R deletion on liver in middle-aged mice on STD and HFD diet. (A) Effects of IGF1R deletion on liver weight; and (B) on liver triglyceride content in males and females fed with either a STD or HFD and compared to young (13 weeks-old) control (Igf1rfl/fl ) male and females on STD. Values are means ± SEM of 4-11 animals per group. aap < 0.01 and aaap < 0.001 vs. young Igf1rfl/fl mice. *p < 0.05, **p < 0.01 vs. middle-aged Igf1rfl/fl STD animals. [file Image_3.tif]
