# Supplementary material for: IGF1R is a mediator of sex-specific metabolism in mice: Effects of age and high-fat diet
Source: Front Endocrinol (Lausanne). 2022 Oct 20;13:1033208. doi: 10.3389/fendo.2022.1033208 (PMC9638844; doi:10.3389/fendo.2022.1033208)
Supplement: Supplementary Table 1 — Primers sequences used for real-time PCR. [file Table_1.docx]

**Supplementary Table 1**: Primers sequences used for real-time PCR

| **Gene** | **Metabolic pathway** | **Forward** | **Reverse** |
| --- | --- | --- | --- |
| ***Igf1*** | IGF signaling | CAGAAGCGATGGGGAAAAT | GTGAAGGTGAGCAAGCAGAG |
| ***Igfbp2*** | IGF signaling | GGGAGTGCTGGTGTGTGA | CTGCTGGTGTTCGGGATG |
| ***Igfbp3*** | IGF signaling | GCCCTCTGCCTTCTTGATTT | TCACTCGGTTATGGGTTTCC |
| ***Ucp2*** | Mitochondrial proton leak | AAGGGGAGAGTCAAGGGCTA | ATTGTAGAGGCTGCGTGGAC |
| ***Cpt1a*** | FA β-oxidation | CCAGGCTACAGTGGGACATT | GAACTTGCCCATGTCCTTGT |
| ***Fas*** | Lipogenesis | TGGGTTCTAGCCAGCAGAGT | ACCACCAGAGACCGTTATGC |
| ***F4/80*** | Macrophage marker | CTTTGGCTATGGGCTTCCAGTC | GCAAGGAGGACAGAGTTTATCGTG |
| ***Cd68*** | Macrophage marker | CTTCCCACAGGCAGCACAG | AATGATGAGAGGCAGCAAGAGG |
| ***Tnfα*** | Inflammation | ACGGCATGGATCTCAAAGAC | AGATAGCAAATCGGCTGACG |
| ***β-actin*** | Housekeeping gene | GGCTGTATTCCCCTCCATCG | CCAGTTGGTAACAATGCCATGT |

FA: fatty acid
